# Discovery of nonautonomous modulators of activated Ras

**DOI:** 10.1093/g3journal/jkac200

**Published:** 2022-08-05

**Authors:** Marcos Corchado-Sonera, Komal Rambani, Kristen Navarro, Raleigh Kladney, James Dowdle, Gustavo Leone, Helen M Chamberlin

**Affiliations:** Department of Molecular Genetics, Ohio State University, Columbus, OH 43210, USA; Department of Cancer Biology and Genetics, Ohio State University, Columbus, OH 43210, USA; Biomedical Sciences Graduate Program, Ohio State University, Columbus, OH 43210, USA; Department of Molecular Genetics, Ohio State University, Columbus, OH 43210, USA; Department of Cancer Biology and Genetics, Ohio State University, Columbus, OH 43210, USA; Department of Cancer Biology and Genetics, Ohio State University, Columbus, OH 43210, USA; Department of Molecular Genetics, Ohio State University, Columbus, OH 43210, USA; Department of Cancer Biology and Genetics, Ohio State University, Columbus, OH 43210, USA; Department of Molecular Genetics, Ohio State University, Columbus, OH 43210, USA

**Keywords:** cell communication, Ras, ATP synthase, *Caenorhabditis elegans*, vulval development

## Abstract

Communication between mesodermal cells and epithelial cells is fundamental to normal animal development and is frequently disrupted in cancer. However, the genes and processes that mediate this communication are incompletely understood. To identify genes that mediate this communication and alter the proliferation of cells with an oncogenic Ras genotype, we carried out a tissue-specific genome-wide RNAi screen in *Caenorhabditis elegans* animals bearing a *let-60(n1046*gf*)* (RasG13E) allele. The screen identifies 24 genes that, when knocked down in adjacent mesodermal tissue, suppress the increased vulval epithelial cell proliferation defect associated with *let-60(n1046*gf*).* Importantly, gene knockdown reverts the mutant animals to a wild-type phenotype. Using chimeric animals, we genetically confirm that 2 of the genes function nonautonomously to revert the *let-60(n1046*gf*)* phenotype. The effect is genotype restricted, as knockdown does not alter development in a wild type (*let-60(+)*) or activated EGF receptor (*let-23(sa62*gf*)*) background. Although many of the genes identified encode proteins involved in essential cellular processes, including chromatin formation, ribosome function, and mitochondrial ATP metabolism, knockdown does not alter the normal development or function of targeted mesodermal tissues, indicating that the phenotype derives from specific functions performed by these cells. We show that the genes act in a manner distinct from 2 signal ligand classes (EGF and Wnt) known to influence the development of vulval epithelial cells. Altogether, the results identify genes with a novel function in mesodermal cells required for communicating with and promoting the proliferation of adjacent epithelial cells with an activated Ras genotype.

## Introduction

Communication between mesodermal cells and epithelial cells is an important process required for normal development and physiology of animals, such as during gastrulation of vertebrates and invertebrates, lung branching morphogenesis, and intestinal crypt homeostasis (Brügger *et al.* 2020; Iber 2021; John and Rauzi 2021). This communication also is important in altered physiological conditions such as wound healing, organ regeneration, and pregnancy and is often disrupted in reproducible ways in disease conditions, such as cancer (Nelson and Bissell 2006). For example, mesodermally derived cancer-associated fibroblasts represent a major component of the microenvironment in epithelial-derived solid tumors and can influence cancer progression by altering properties of the cancer cells, such as proliferation, malignancy, therapeutic resistance, and metastasis (Paulsson and Micke 2014; Farhood *et al.* 2019; Alexander and Cukierman 2020). How mesodermal–epithelial interactions differ between normal and diseased states, and the molecular factors responsible for these effects, are poorly understood.

**Fig. 1.**
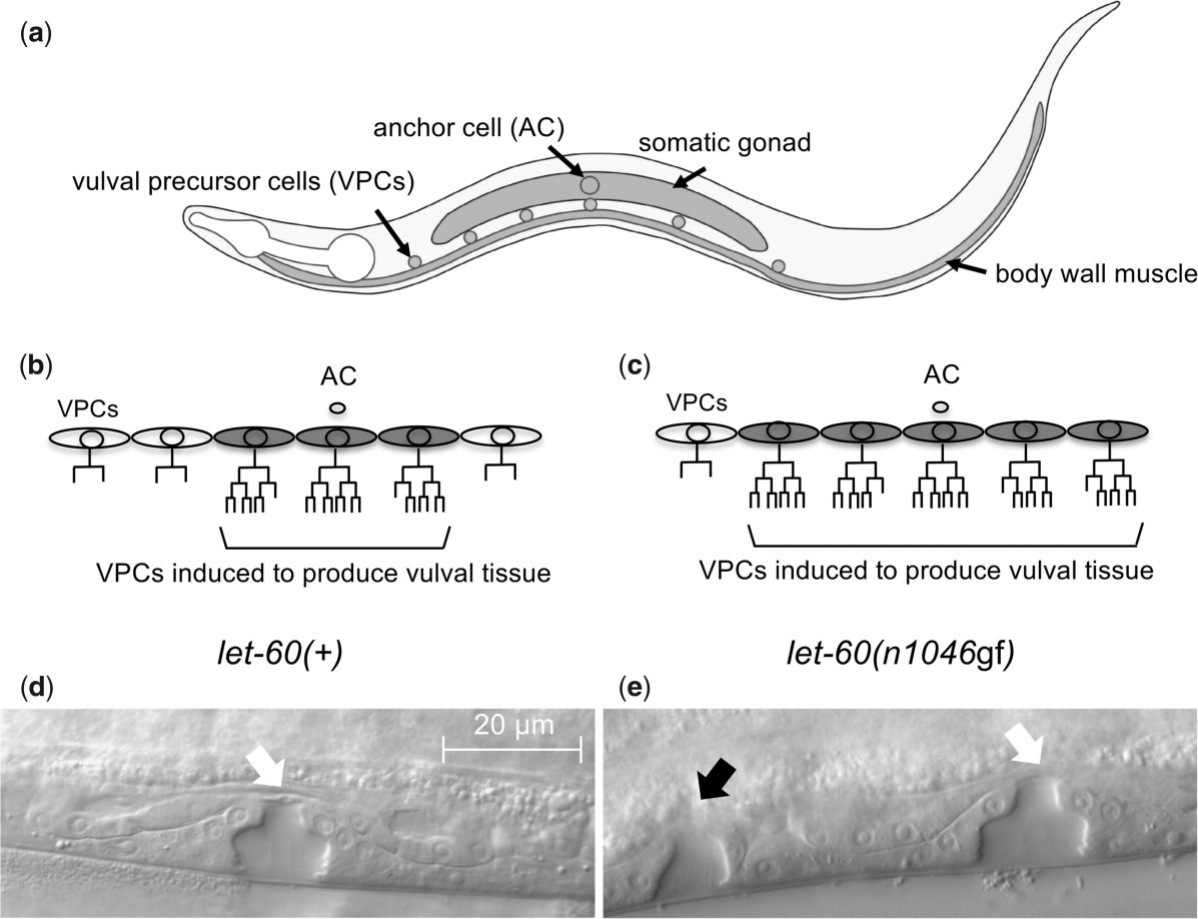
Vulval development in *C. elegans*. a) A cartoon of a *C. elegans* hermaphrodite in the third larval (L3) stage, illustrating the relative position of VPCs and some adjacent mesodermal cells: somatic gonad (including the AC) and (ventral) body wall muscle. VPC lineages in wild type (*let-60(+)*) (b) and a representative animal with an activated Ras genotype (*let-60(n1046*gf*)*) (c). Normally, through coordination of several cell signals, 3 of 6 VPCs are “induced” to divide and produce cells of the vulva. In most *let-60(n1046*gf*)* animals, more than 3 (and up to 6) VPCs divide to produce vulval cells. Mid-body region of fourth larval stage (L4) hermaphrodites, identifying the vulval structures produced from VPC descendants in *let-60(+)* (d) and *let-60(n1046*gf*)* (e) animals. A single vulval structure forms from the offspring of 3 induced VPCs (white arrow), and additional structures result if ectopic VPCs divide to produce vulval cells (black arrow).

To uncover novel mesoderm-derived factors that mediate communication with epithelial cells in vivo, we utilize the well-defined vulval development process in the nematode, *Caenorhabditis elegans*, where signals from the mesoderm coordinate the spatial patterning of the epithelial vulva precursor cells (VPCs; Fig. 1). In these animals, normal vulval development results when 3 of 6 VPCs are “induced” to divide and produce vulval cell types in response to the LIN-3/EGF signal from the anchor cell (AC), part of the somatic gonad (Hill and Sternberg 1992). LIN-3/EGF acts through a canonical Ras-MAPK pathway, which includes the Ras protein LET-60. *Ras* is a well-characterized oncogene, and hyperactive mutations in the *C. elegans let-60/Ras* gene likewise leads to increased epithelial proliferation in worms, resulting in the induction of more than 3 VPCs and the production of ectopic vulval tissue, identified as the Multivulva, or Muv, phenotype (Beitel *et al.* 1990; Fig. 1). Hyperactive alleles of *let-60*, notably the *let-60(n1046*gf*)* RasG13E missense allele, have long provided a simplified genetic framework to evaluate functions of oncogenic Ras, and have been utilized to identify and characterize components of the Ras-MAPK signaling pathway (Wu and Han 1994; Kornfeld *et al.* 1995; Singh and Han 1995; Sundaram and Han 1995; Sternberg and Han 1998; Reiner *et al.* 2008). The combination of a well-defined developmental system and the capacity to genetically and/or epigenetically manipulate the system in a controlled manner offers a powerful framework to identify new genes that influence communication between tissue types under both normal and genetically perturbed conditions.

Here, we utilize a genome-wide, tissue-specific RNAi strategy to identify novel genes that modulate mesoderm-to-epithelial signaling in *C. elegans* animals bearing an activated Ras genotype, *let-60(n1046*gf*)*. We identify 24 genes that, when knocked down in mesodermal cells, revert the Muv phenotype. Notably, knockdown reverts the Muv phenotype without disrupting the capacity of the VPCs to produce normal vulval tissue. Likewise, knockdown does not disrupt vulval development in a *let-60(+)* or *let-23/EGFR(gf)* genetic background, nor the general developmental or functional aspects of the mesodermal tissues, although the genes encode proteins involved in essential processes such as histones, ribosomes and mitochondrial ATP synthases. Using genetically chimeric animals, we confirm the nonautonomous role for 2 genes (*szy-5* and *hpo-18*) encoding a zinc finger protein and an ATP synthase. By comparing the effect of *szy-5* and *hpo-18* disruption to 2 mesodermal signal types important for vulval development, LIN-3/EGF and WNT, we show that the identified genes alter Ras signaling in ways distinct from other signaling pathways, arguing that different mesodermal signals required for supporting the inappropriate proliferation of adjacent epithelial cells are sensitive to genotype. Altogether, the results identify a novel function for mesodermal cells in communicating with epithelial cells that is uncovered in animals bearing an activated Ras mutation.

## Materials and methods

### Worm maintenance and genetics

*C. elegans* strains were grown on NGM plates seeded with *Escherichia coli* OP50 as a food source, using established methods (Stiernagle 2006). The wild-type *C. elegans* is N2 Bristol. Specific strains and genotypes used are listed in Supplementary Table 1. The deletion allele of *C18B12.6(gu262)* was generated using the CRISPR method and the plasmids of Dickinson *et al.* (2015), with the modification that 5′ and 3′ homology arms were selected to flank the coding exons of the ERGIC2-related gene *C18B12.6*. Consequently, introduction of the repair template replaces the gene with sequences of the vector, including the hygromycin resistance and *sqt-1(d)* markers of the self-excising cassette. The allele *gu262* was produced by removal of the self-excising cassette as described in Dickinson *et al.* (2015). Primers used to produce the plasmids and genotype the allele are listed in Supplementary Table 2.

### RNAi screen and RNAi knockdown for phenotypic evaluation

For the genetic screen and subsequent RNAi analyses, we utilized the mesodermal-RNAi and VPC-RNAi genetically engineered systems reported previously (Liu *et al.* 2017). In *C. elegans*, RNAi precursor molecules move freely across tissues, which permits gene knockdown by the introduction of double-stranded RNA by feeding or soaking, but also means that the effect of double-stranded RNA expressed in 1 cell type is not restricted to that cell type (Jose and Hunter 2007). Thus, we utilized a method that restricts RNAi sensitivity and response by restoring activity of the *rde-1* (RNAi-Defective-1) gene to specific cells (Tabara *et al.* 1999; Qadota *et al.* 2007). We generated a transgene with *rde-1(+)* cDNA expressed under the control of 3 different promoters that express in mesodermal tissues [*guIs37*; includes *myo-3::rde-1(+)*, *ddr-2::rde-1(+)*, and sequences from *lin-3* that drives expression in the AC (*ACEL::Δpes-10::rde-1(+)*)]. This transgene was introduced into animals of genotype *rrf-3(pk1426)*; *rde-1(ne219)*, where the transgene restores RNAi activity into body wall muscle and somatic gonad tissues, and *rrf-3(pk1426)* enhances the RNAi effect in cells capable of the response (Simmer *et al.* 2002). Gene knockdown via RNAi in these animals is restored in mesodermal tissues, and the VPCs are defective in the RNAi response, as demonstrated by knockdown of *lin-3/EGF* (required in mesoderm) and *lin-39/Hox* (required in VPCs) (Liu *et al.* 2017). *let-60(n1046*gf*)* was crossed into this genetic background, producing the strain CM2453 (mesodermal-RNAi strain). A similar transgene system in which RNAi sensitivity is restored only in the VPCs using *lin-31*-derived regulatory sequences (*guIs39*) was likewise crossed with *let-60(n1046*gf*)* to produce the strain CM2731 (VPC-RNAi strain), used to produce the results of Supplementary Fig. 3.

The genome-wide screen was performed using the Ahringer library (Source Bioscience LifeSciences), as in Liu *et al.* (2017). Briefly, fresh overnight bacteria cultures containing each RNAi clone were seeded onto NGM plus 1 mM IPTG and 25 μg/ml carbenicillin in either 12-well trays or individual 35-mm Petri plates. Synchronized embryos (or L1 larvae for subsequent analyses) were seeded onto these plates and incubated for an appropriate time at 20°C. For the primary RNAi screen, eggs were plated and, 4 days later, approximately 50 animals were scored for presence of the Muv phenotype using a dissecting microscope. Each clone was tested in 3 independent replicates, and clones that reduced the Muv phenotype by 30% or more, compared to the control (empty vector, L4440) strain scored on the same day. To confirm reproducibility of the knockdown phenotype, an independent test of the 47 genes recovered from this initial screen was carried out, and clones conferring less than 50% Muv phenotype (compared to 70% or greater for control) were retained, and subjected to Sanger sequencing to confirm the gene targeted by the plasmid. The resulting 24 genes are listed in Table 1.

**Table 1.** Twenty-four genes identified as modulators of the activated Ras (*let-60(n1046*gf*)*) Muv phenotype.

WormBase sequence ID	Gene	Human ortholog	Description of human ortholog	**% Muv** ^a^
Histone genes				
F45E1.6	*his-71*	H3F3B	H3 histone family member 3B	31
B0035.9	*his-46*	HIST1H4G	Histone cluster 1 H4 family member g	20
F08G2.2	*his-43*	HIST2H2AB	Histone cluster 2 H2A family member b	16
T10C6.12	*his-3*	HIST2H2AB	Histone cluster 2 H2A family member b	21
F17E9.10	*his-32*	HIST2H3D	Histone cluster 2 H3 family member d	20
Ribosome genes				
C04F12.4	*rpl-14*	RPL14	Ribosomal protein L14	5
K11H12.2	*rpl-15*	RPL15	Ribosomal protein L15	18
F55D10.2	*rpl-25.1*	RPL23A	Ribosomal protein L23a	11
W01D2.1	W01D2.1	RPL37	Ribosomal protein L37	16
C37A2.7	C37A2.7	RPLP2	Ribosomal protein lateral stalk subunit P2	27
Mitochondrial ATP synthase genes				
F32D1.2	*hpo-18*	ATP5F1E	ATP synthase F1 subunit epsilon	9
C06H2.1	*atp-5*	ATP5PD	ATP synthase peripheral stalk subunit d	16
T05H4.12	*atp-4*	ATP5PF	ATP synthase peripheral stalk subunit F6	13
Other genes				
C18B12.6	C18B12.6	ERGIC2	ERGIC and golgi 2	35
F36A2.8	*phip-1*	PHPT1	Phosphohistidine phosphatase 1	18
H17B01.1	*fgt-1*	SLC2A3	Solute carrier family 2 member 3	27
F40F11.2	*mig-38*	BICRAL	BRD4 interacting chromatin remodeling complex associated protein like	29
W04A8.1	W04A8.1	MCPH1	Microcephalin 1	23
D1007.4	D1007.4	GEMIN6	Gem nuclear organelle protein 6	27
C27A12.2	*szy-5*	ZNF709	Zinc finger protein 791	26
Nematode-specific genes				
F17B5.5	*clec-110*	None	C-type LECtin	14
K09E3.5	*lido-5*	None	LIn-8 Domain containing	23
F55D12.1	F55D12.1	None	N/A	44
W04G3.8	*lpr-3*	None	LiPocalin-Related protein	26
Negative control				
Empty plasmid (L4440)				70

a All experimental values statistically different from negative control (*P* < 0.05, proportional 2-tailed *Z* test, with Bonferroni correction). *N* ≥ 40 for each condition, except for *his-32* and *rpl-14* (full data and sample numbers are provided in Supplementary tables).

### Genetic chimeras

Genetic chimeras were generated using the method of Artiles *et al.* (2019). *let-60(n1046*gf*)*, *let-60(n1700*gf*)*, or *let-23(sa62*gf*)* were crossed into strains bearing transgene *ccTi1594* (which causes over-expression of GPR-1 in the germline) to produce strains CM2780 (*ccTi1594 umnIs7; let-60(n1046*gf*)*, maternal chimera strain with *myo-2::GFP*), CM2783 (*ccTi1594 unc-119(ed3); hjSi20 let-60(n1046*gf*)*, maternal chimera strain with *myo-2::mCherry*), CM2899 (*ccTi1594 unc-119(ed3); hjSi20 let-60(n1700*gf*)*, maternal chimera strain with *myo-2::mCherry*), and CM2868 (*let-23(sa62*gf*); ccTi1594 unc-119(ed3); hjSi20*, maternal chimera strain with *myo-2::mCherry*). These strains were used as the maternal strains for crosses with paternal control [VS21 (*hjSi20, myo-2::mCherry*), CGC18 (*umnIs7*, *myo-2::GFP*), or N2 Bristol (wild type)] and genetic mutant strains. Chimeric animals in which the maternal genome segregates into AB (and paternal into P1) exhibit a characteristic pattern of maternal fluorescence in the anterior pharynx that is easy to identify under a dissecting microscope (Artiles *et al.* 2019). At lower frequency, we also observed animals with maternal genome in P1 (and paternal in AB), as well as standard heterozygotes, but we selected only the AB maternal/P1 paternal class of animals for analysis as they provide the desired genotypes in the relevant cells. Homozygous male parents were crossed with the maternal strain and chimeric animals with maternal DNA restricted to AB were selected as larvae for either direct evaluation of vulval development, or removal to a separate plate for evaluation of adult characteristics (Muv phenotype). Mutations for which homozygous animals are inviable or sterile were crossed in as balanced heterozygotes, utilizing a balancer labeled with a *myo-2::gfp* or *myo-2::mCherry* reporter. The maternal strain was selected to have a fluorescent marker in contrast with the balancer, and chimeric offspring with characteristic maternal fluorescence but lacking fluorescence from the paternal genome (nonbalancer) were selected for analysis. A control cross (typically using N2 wild-type males) was included for each experiment, to control for possible variation in the phenotype of the maternal strain. While chimeric animals that received a paternal balancer were observed among the cross, these animals were not used as a control as it was observed (notably for *nT1(qIs51)*) that at least some of the mutations on the balancer chromosome itself limit the viability of chimeric animals and can modulate the Muv phenotype of *let-60(n1046*gf*)* (data not shown).

### Microscopy

**Fig. 2.**
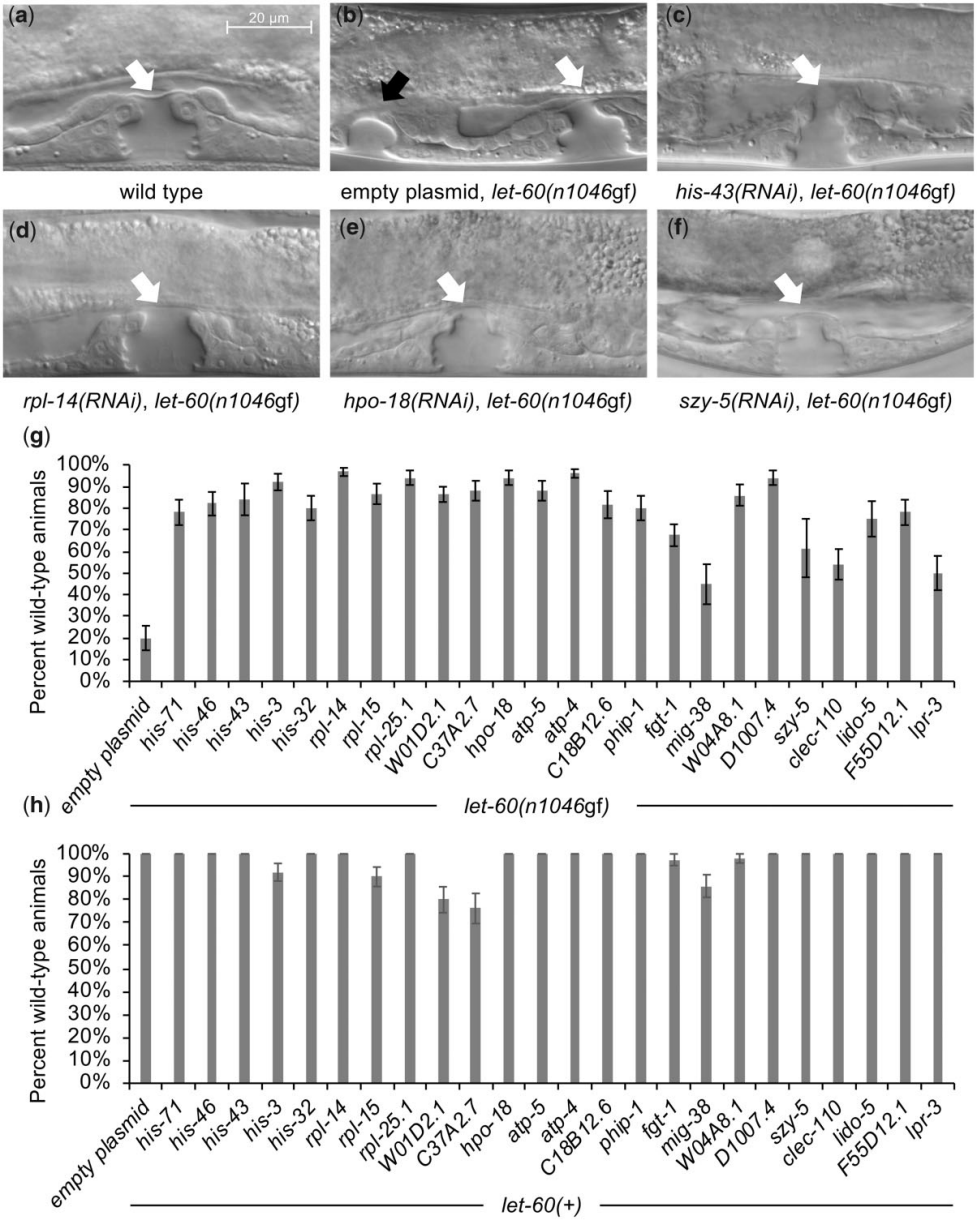
Gene knockdown reverts *let-60(n1046*gf*)* to a wild-type phenotype but does not disrupt vulva development in a *let-60(+)* background. a–f) DIC images of the ventral epidermis in L4 stage. a) In wild-type animals, the offspring of 3 ventral epithelial cells (VPCs) organize to form a single vulval opening, identified with a white arrow, as in Fig. 1. b) *let-60(n1046*gf*)* mutants bearing the mesodermal-RNAi system, treated with control RNAi, produce a Muv phenotype with a primary vulval opening (white arrow) as well as ectopic vulval tissue (black arrow) resulting from the division of additional VPCs. c–f) Images of mesodermal-RNAi knockdown of representative genes. RNAi knockdown reverts the *let-60(n1046*gf*)* Muv to a wild-type phenotype. Uncropped images are provided in Supplementary Fig. 1. g) Mesodermal-RNAi knockdown of all genes identified in the screen revert *let-60(n1046*gf*)* to a wild-type phenotype. Data are indicated as % wild type (rather than % Muv, as in Table 1) to highlight this reversion to wild type effect. In both control and experimental knockdown, the nonwild-type animals were predominantly Muv (as is also seen in chimeras, Fig. 4). The empty plasmid is L4440. *n* ≥ 40 for each RNAi knockdown except for *his-43*, C18B12.6, *mig-38*, *szy-5*, *lido-5* (full data and sample numbers provided in Supplementary tables). All experimental values statistically different from negative control, except for *mig-38*, *szy-5*, and *lpr-3* (*P* < 0.05, 2-tailed proportional *Z* test, with Bonferroni correction). h) Mesodermal-RNAi knockdown of all genes identified in the screen have minimal effect on vulval development in a *let-60(+)* background. Knockdown of some genes results in a low percentage (<25%) of animals with nonwild-type vulva development. These abnormal animals include a mixture of abnormal vulva morphology, and possible reduced vulva induction, but no animals were vulvaless (Vul). Error bars correspond to standard error. *n* ≥ 40 for each RNAi knockdown except for *his-32*, *fgt-1*, *szy-5*, *lido-5*, and *lpr-3* (full data and sample numbers provided in Supplementary tables).

Vulval cell morphology and development were evaluated in L4 animals using DIC optics at 100× magnification (Figs. 2, 4, and 7), while the VPCs are dividing or have completed their divisions and the cells are initiating morphogenesis as done previously (Chamberlin *et al.* 2020). Gonad morphology (Fig. 5) was likewise evaluated in L4 animals using DIC optics at 100× magnification. LAM-1::mCherry localization (Fig. 6) was observed in L3 animals under both DIC and fluorescent imaging at 100× magnification. Stage of development was assessed first based on the (inferred) number of divisions completed by the P6.p cell, and then fluorescent images were collected using a constant exposure time. For RNAi experiments, sibling animals were raised to adulthood and evaluated for Muv phenotype, to confirm the efficacy of gene knockdown.

### MitoTracker staining and quantification

We evaluated the presence and distribution of mitochondria in the AC following the method of Kelley *et al.* (2019). Briefly, 200 μl of 250 nM MitoTracker Red CMXRos Probes diluted in M9 buffer was added to each RNAi plate 1 h prior to plating synchronized L1-stage animals and incubated for 32 h at 20°C to obtain L3-stage animals. Stage of development was assessed first based on the (inferred) number of divisions completed by the P6.p cell, and then, fluorescent images were taken using a constant exposure time. For polarity calculations, ImageJ software (https://imagej.nih.gov/ij/index.html; Rasband 1997) was used to draw 2 regions of interest within the cell, the basal half and the apical half. Mean fluorescence intensities were measured for each half and background fluorescence intensities were measured by selecting a 5-pixel diameter circle of nonfluorescent region within each half. To calculate polarity, background fluorescence intensities were subtracted from mean fluorescence intensities and the ratio of basal fluorescence to apical fluorescence was calculated. Sibling animals were raised to adulthood and evaluated for Muv phenotype, to confirm the efficacy of gene knockdown.

### Locomotion assay

We evaluated muscle function using the body bends per minute assay, as in Sawin *et al.* (2000). Ten L4-stage animals were selected per RNAi knockdown condition and observed at room temperature using a dissecting microscope. Prior to collecting data, animals were transferred from plates containing RNAi bacteria to fresh plates seeded with OP50 *E. coli* bacteria. Animals were allowed to move freely for 1 min and body bends were counted as described in (Sawin *et al.* 2000) for 2 min immediately after. Sibling animals were raised to adulthood and evaluated for Muv phenotype, to confirm the efficacy of gene knockdown.

## Results

### A genome-wide RNAi screen identifies nonautonomous modulators of activated LET-60/Ras

To identify genes that modulate the activity of activated LET-60/Ras in a nonautonomous manner, we utilized a genetically engineered mesodermal-RNAi system, in which the RNAi response is limited to mesodermal cells (somatic gonad and body wall muscle; Fig. 1) by introducing *rde-1(+)* under the control of tissue-restricted promoters in *rde-1* mutant animals (Liu *et al.* 2017). We have previously validated this system using knockdown of *lin-3/EGF* (required in mesoderm) and *lin-39/Hox* (required in VPCs), and used this system to identify genes that, when knocked down, behave in a tumor-suppressor-like manner and enhance VPC proliferation in mutants lacking *gap-1* (Liu *et al.* 2017). To identify mesodermal genes that normally promote VPC proliferation (have oncogene-like properties), we crossed this system into a strain homozygous for the *let-60(n1046*gf*)* allele (Beitel *et al.* 1990). We systematically treated animals from this strain with *E. coli* clones from the Ahringer RNAi library, which includes bacterial strains that individually target roughly 86% of genes in the *C. elegans* genome (Kamath *et al.* 2003). We identified 24 genes that, when knocked down, reliably suppressed the *let-60(n1046*gf*)* phenotype and reduced the proportion of adult Muv animals from 70% to 85% to less than 50% of animals (Table 1). Multiple of the identified genes fell into 3 main categories based on the general function of their protein products: histones, ribosomes, mitochondrial ATP synthases. Overall, 83% (20/24) of the genes have clear human orthologs, arguing that the screen identified conserved modulators of Ras signaling.

To identify the nature of the effect mediated by these genes, we evaluated the morphology of the developing vulva in the fourth larval stage (L4) of animals treated with mesodermal-RNAi (Fig. 2). In animals bearing the *let-60(n1046*gf*)* allele, we found that the proportion of animals with a wild-type vulva morphology is notably increased in response to knockdown of each of the genes identified in the screen (Fig. 2g), indicating that the general effect is to restore normal development, rather than to interfere with or block the production of vulval cells. Knockdown of each gene in a *let-60(+)* genetic background had minimal effect on vulval development (Fig. 2h), arguing the role for these genes is sensitive to the *let-60(n1046*gf*)* mutant condition, rather than performing a role in normal vulval development. Finally, knockdown within the VPCs themselves is not sufficient to restore normal development (Supplementary Fig. 2), supporting the idea that the genes function in a nonautonomous manner.

### Genetic mutants validate genes from the mesodermal-RNAi screen

**Fig. 3.**
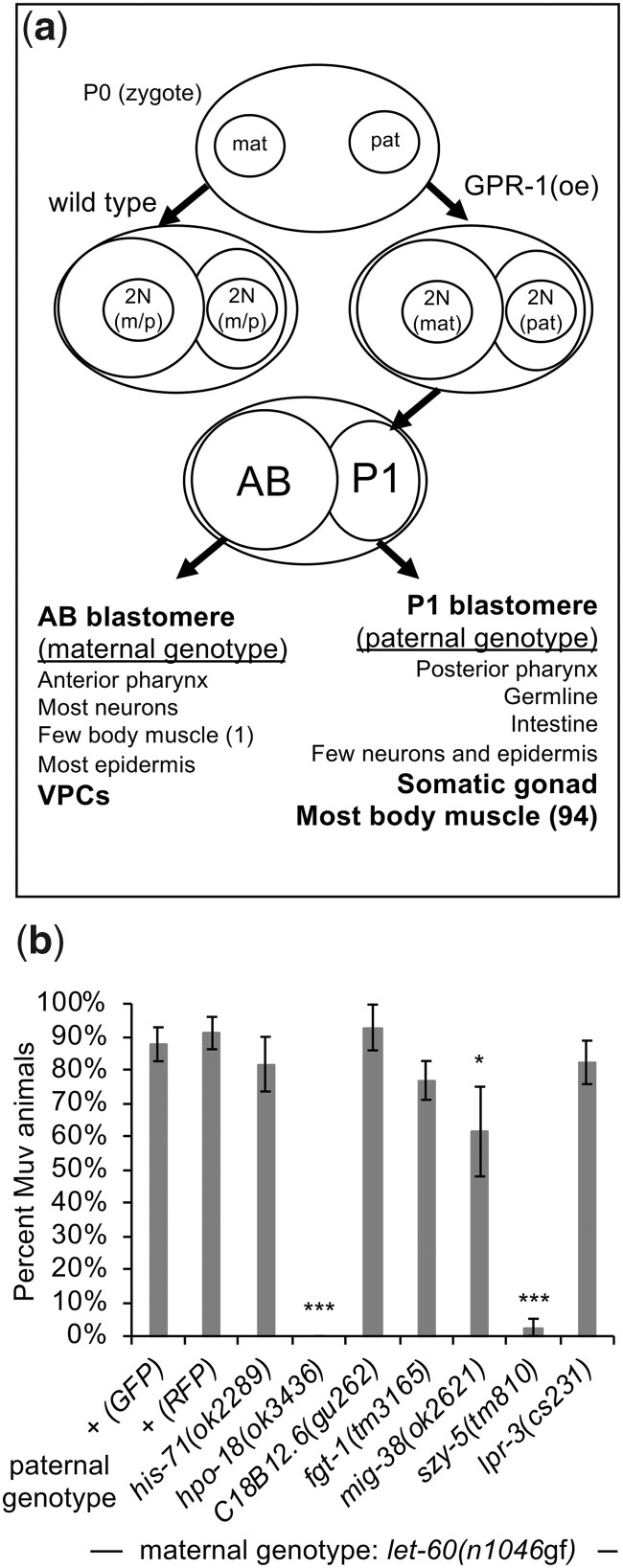
Genetic chimeras demonstrate that *hpo-18* and *szy-5* act nonautonomously to revert the *let-60(n1046*gf*)* phenotype. a) Schematic representation of the genetic chimera method, after Artiles *et al.* (2019). GPR-1 is overexpressed (GPR-1(oe)) in the maternal germline and oocytes, causing the pronuclei to segregate to daughter cells without fusing in the zygote. The chimera class evaluated in this experiment is the case where replicated maternal chromosomes segregate to the AB blastomere (precursor to the VPCs), while replicated paternal chromosomes segregate to the P1 blastomere (precursor to most mesodermal cells). b) Chimeric animals with paternal (P1) mutant alleles for each of 7 genes (with *let-60(+)*) in the P1 cell lineage and maternal *let-60(n1046*gf*)* (with wild type for each paternal gene) in the AB cell lineage. Alleles of *szy-5* and *hpo-18* confer a strong suppression of the Muv phenotype. *ril-1(ok2492)* and the strain VC2839 (with the *ok2678* allele that deletes a portion of *atp-4* plus the adjacent gene *T05H4.11*) were also tested, but resulted in chimeras that do not survive to adulthood. Animals with *mig-38(ok2621)* exhibit a modest modulatory effect in adults, but subsequent analysis of L4 chimeric animals suggest the phenotype derives from delayed development or altered morphogenesis rather than reduced vulval development (data not shown). + (GFP) and + (RFP) represent 2 control experiments, using a paternally supplied *myo-2::GFP* or *myo-2::mCherry/RFP* transgene (*umnIs7* or *hjSi20*, respectively), to contrast with the fluorescent marker in the maternal strains, as in Artiles *et al.* (2019). These paternal genotypes are wild type except for the transgene. Error bars correspond to standard error. Asterisks indicate statistically different from control (*Z*-test, *P < 0.05; ***P < 0.001). Full data and sample numbers are provided in Supplementary tables.

**Fig. 4.**
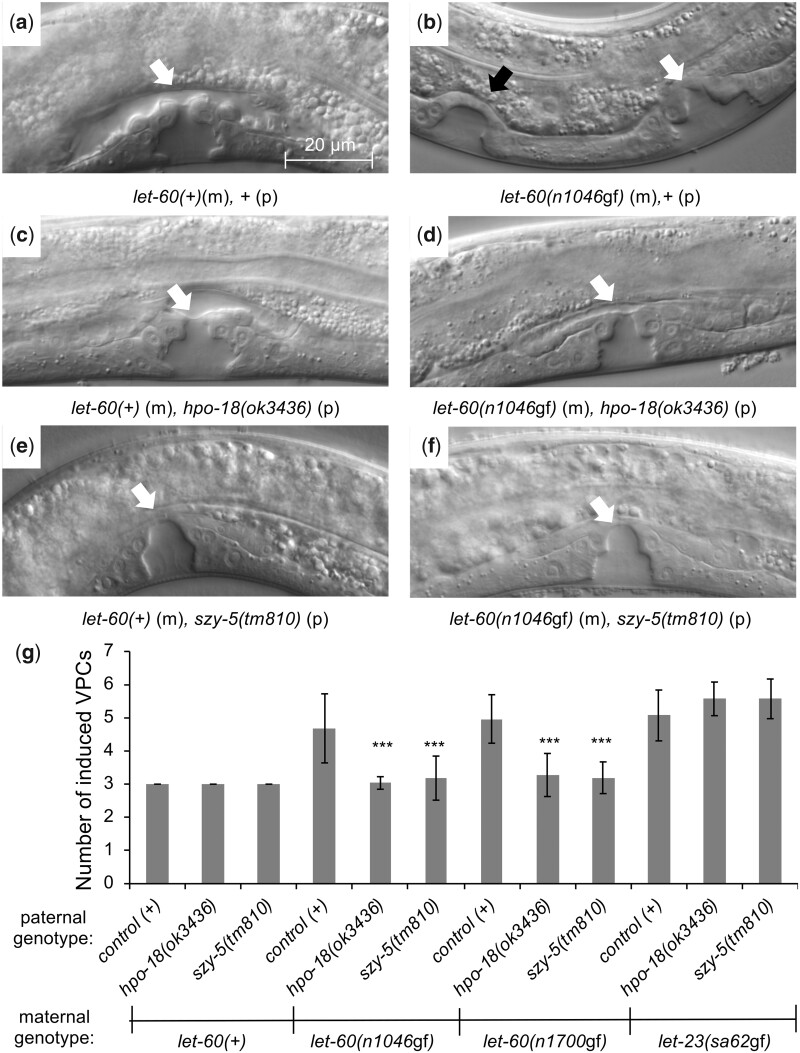
Loss of *hpo-18* or *szy-5* in the P1 lineage does not disrupt normal vulva development and revert *let-60(*gf*)* to a wild-type phenotype. a–f) DIC images of the ventral epidermis in L4 stage, as in Fig. 2. Chimeric animals generated as in Fig. 3. Genotype of animal in each image indicated, with (m) identifying the relevant maternally derived genotype, and (p) the relevant paternally derived genotype. a, b) Control chimeric animals, showing wild-type vulval morphology in *let-60(+)* and ectopic vulval tissue in *let-60(n1046*gf*).* c–f) Animals homozygous for *hpo-18(ok3436)* or *szy-5(tm810)* in P1 exhibit normal vulval morphology when AB genotype is *let-60(+)* and revert *let-60(n1046*gf*)* to a wild-type phenotype. g) Quantification of the effect. In wild-type animals, 3 of 6 VPCs divide to produce vulval tissue and are identified as induced to produce vulval tissue, and this is unaltered in *hpo-18(ok3436)* or *szy-5(tm810)* chimeras. In animals with maternally provided *let-60(n1046*gf*)*, *let-60(n1700*gf*)*, or *let-23(sa62*gf*)* in AB, more than 3 VPCs divide and are induced (detailed in Fig. 1). This phenotype is suppressed to wild type if mutations in *hpo-18* or szy-5 are present in P1 for both *let-60* alleles, but not for *let-23(sa62*gf*)*. Error bars correspond to standard deviation. Asterisks indicate statistically different from control (*t*-test, ***P < 0.001). All conditions include 21 or more animals. Full data and sample numbers provided in Supplementary tables.

To validate the results of the RNAi screen, we utilized the genetic chimera method of (Artiles *et al.* 2019). This method produces animals with a distinct genotype in cells derived from the embryonic AB blastomere (which includes the VPCs) from cells derived from the P1 blastomere (which includes the somatic gonad and most of the body wall muscle cells). It therefore both tests the capacity of a mutant allele to replicate the RNAi knockdown effect, as well as validates the nonautonomous nature of the modulation, as the relevant *let-60* genotype and the relevant genetic mutation are restricted to distinct larval cells based on their embryonic origin. Using this method, we selected animals with *let-60(n1046*gf*)* in AB-derived cells, and with *let-60(+)* and mutant alleles of genes identified in the RNAi screen in P1-derived cells (see *Materials and Methods*). Mutant alleles were available for 9 of the 24 genes identified in the screen, and P1-chimeric animals using 7 of these alleles survived to late larval or adult stages, permitting phenotypic evaluation. Disruption of 2 of the 7 genes (*hpo-18* and *szy-5*) conferred a strong suppression of the Muv phenotype in adult animals (Fig. 3; reasons why the other mutations fail to suppress are considered in the Discussion). As was observed in animals treated with RNAi, genetic chimeras with mutant *hpo-18* or *szy-5* in P1 revert the defect of *let-60(n1046*gf*)*, and that of an independently isolated RasG13E allele, *let-60(n1700*gf*)*, to wild type (Fig. 4). Importantly, mutation of these genes had no effect on development of wild type (*let-60(+)*) vulval cells. To further assess the specificity of the effect, we asked whether disruption of these genes alters the phenotype associated with a hyperactive allele affecting the EGF receptor gene, *let-23(sa62*gf*).* Like *let-60(*gf*)* mutants, animals homozygous for *let-23(sa62*gf*)* exhibit ectopic vulval tissue and a Muv phenotype due to inappropriate division of more than 3 VPCs (Katz *et al.* 1996). However, animals with *let-23(sa62*gf*)* in AB-derived cells and *let-23(+)* plus *hpo-18(ok3436)* or *szy-5(tm810)* in P1-derived cells exhibit similar increased VPC induction as in control animals (Fig. 4g). This observation is consistent with gene- or allele-restricted roles for the modulators identified in the screen, a property that is also observed for different Ras alleles in human cells (Zafra *et al.* 2020; McFall and Stites 2021). These results genetically validate that loss of *hpo-18* or *szy-5* by RNAi suppresses the Muv phenotype associated with *let-60(n1046*gf*)* and that their role in supporting this phenotype is cell nonautonomous.

### Gross gonad, muscle, and AC functions are maintained in animals subject to mesodermal-RNAi knockdown of the identified genes

**Fig. 5.**
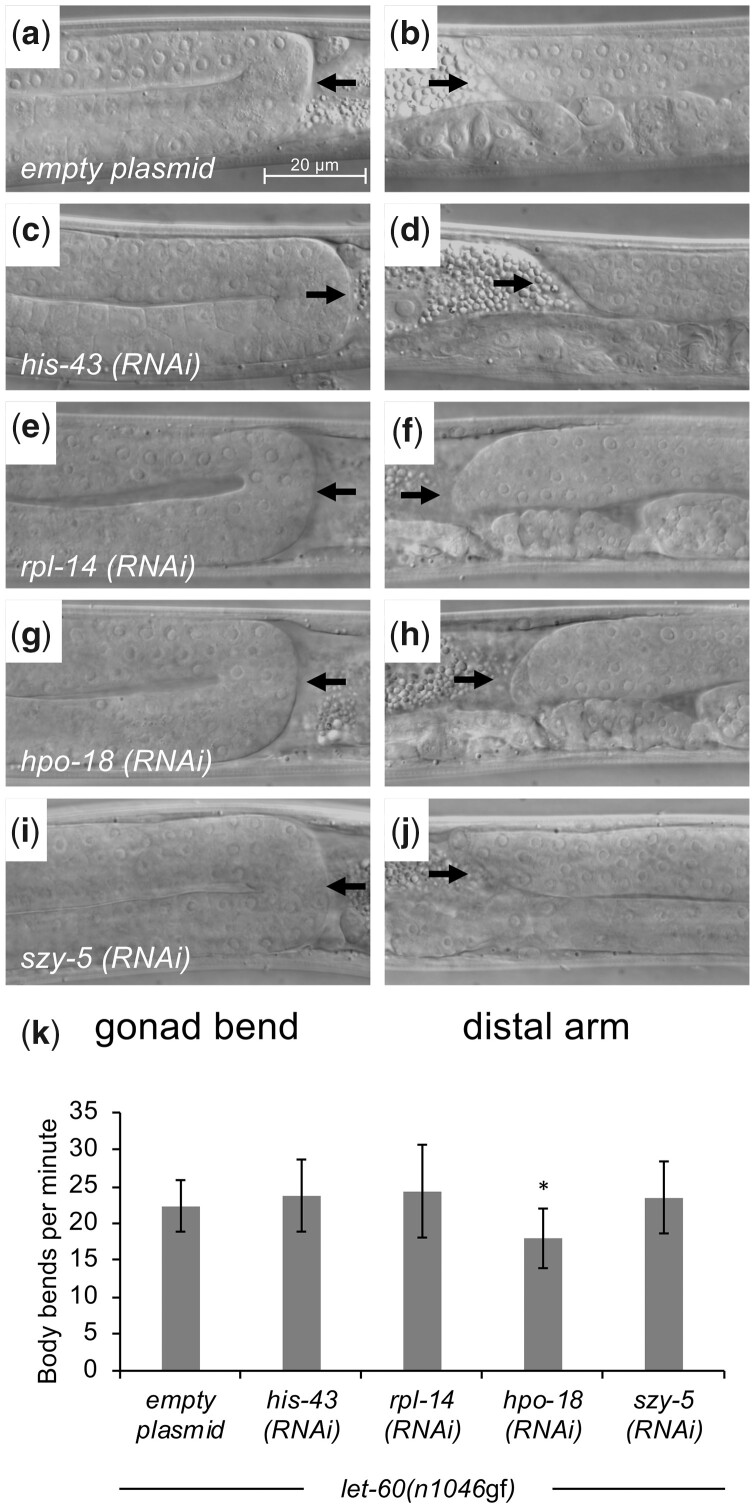
Gross gonad and muscle functions are maintained upon mesodermal-RNAi knockdown of identified genes. a–j) Hermaphrodite animals from CM2453 (mesodermal-RNAi strain) treated with RNAi under conditions that produce a phenotype were selected as L4s and evaluated for gonad morphology, including appropriate bending at the 2 ends of the animal, and presence of the distal ends in the middle of the animal, suggestive of normal somatic gonad anatomy and distal tip cell migration. RNAi knockdown of representative genes identified in the screen do not disrupt normal somatic gonad morphology. Black arrows in the figures from the left column indicate dorsal-to-ventral bend of gonad arm, while black arrows from the right column show presence of a distal gonad arm dorsal to the uterus. Representative animals shown, with *n* = 10 evaluated for each RNAi knockdown and no defects observed. k) Muscle function was evaluated using a locomotion (body bends per minute) assay. RNAi knockdown of representative genes generally does not alter muscle function, although knockdown of *hpo-18* had a modest effect. *n* = 10 for each RNAi knockdown. Error bars correspond to standard deviation. Asterisks indicate statistically different from control (2-tailed *t*-test, *P < 0.05). Raw data provided in Supplementary tables.

Many of the genes identified in the screen encode proteins involved in fundamental cell biological processes, including packaging of DNA, protein translation, and production of ATP. Consequently, we considered the possibility that knockdown of the genes interferes with development or function of the mesodermal cells. To test this hypothesis, we evaluated specific processes that serve as indicators for gross functions of the 3 mesodermal cell types sensitive to gene knockdown by RNAi in our screen: somatic gonad, AC, and muscle. We performed RNAi experiments using the mesodermal-RNAi strain to target the 2 genes validated in the genetic chimera experiment (*hpo-18* and *szy-5*), as well as a representative gene from each of the other main functional classes identified in the RNAi screen: *his-43* and *rpl-14*. Under these conditions, gonad morphology (Fig. 5, a–j) and body wall muscle function were intact (Fig. 5k), although RNAi targeting of *hpo-18* yielded slightly slower-moving animals compared to control animals. Since *hpo-18* encodes a mitochondrial ATP synthase component, we reason that reduction of ATP in the body wall muscle might lead to decreased muscle contractions and movement without impairing muscle tissue integrity.

**Fig. 6.**
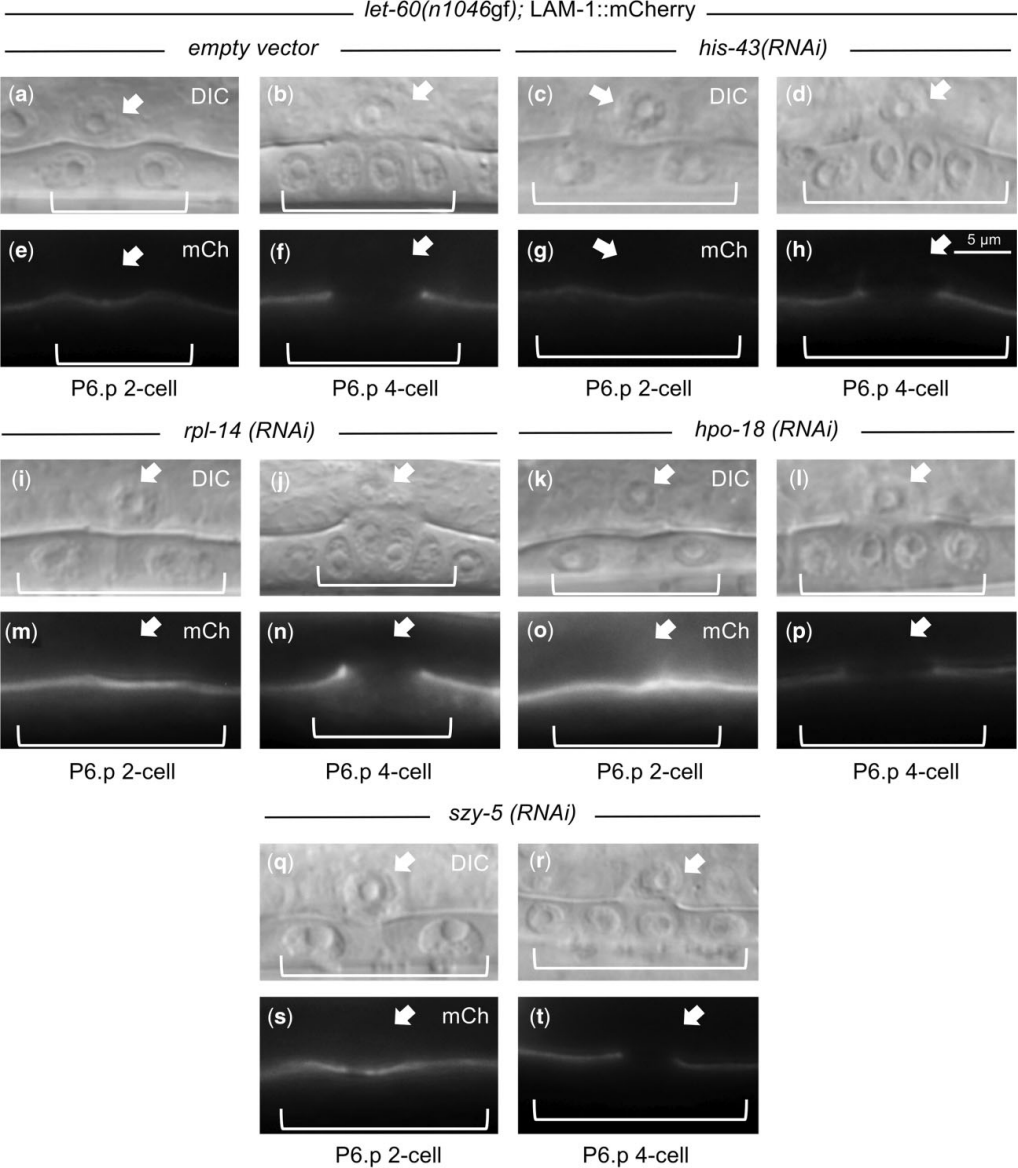
AC anatomy and functions are maintained upon mesodermal-RNAi knockdown of identified genes. Hermaphrodite animals with the mesodermal-RNAi system and a LAM-1::mCherry reporter transgene were treated with RNAi under conditions that produce a phenotype and were selected as L3s and evaluated for AC morphology and capacity to mediate breakdown of the basement membrane between the AC and the dividing VPCs. The presence and timing of basement membrane breakdown (as indicated by clearance of LAM-1::mCherry) between the tissues was maintained under RNAi knockdown of all representative genes from each functional group. *n* = 10 for each RNAi knockdown. Images illustrate representative animals, and no defects were observed.

Finally, we used 3 assays to confirm the AC is functioning as expected when the genes are subject to RNAi knockdown. First, vulval development was normal in all 24 RNAi-targeted conditions (Fig. 2h), arguing that the AC is present and that its function in production of LIN-3/EGF and inducing vulval development is not disrupted. Second, for *hpo-18*, *szy-5*, *his-43* and *rpl-14*, the timing and extent of basement membrane breakdown between the AC and the developing vulva was normal as measured by LAM-1::mCherry removal (Ihara *et al.* 2011) (Fig. 6). Finally, for *hpo-18* and *szy-5*, there was no apparent difference in mitochondria abundance or distribution in the AC as measured by MitoTracker Red to image mitochondria localization (Supplementary Fig. 3). Together, these results indicate gross mesodermal anatomy and functions are maintained under RNAi knockdown conditions sufficient to confer reversion of the *let-60(n1046*gf*)* phenotype.

### Identified genes act in a unique manner, distinct from other mesodermal-derived signals known to influence vulval development

**Fig. 7.**
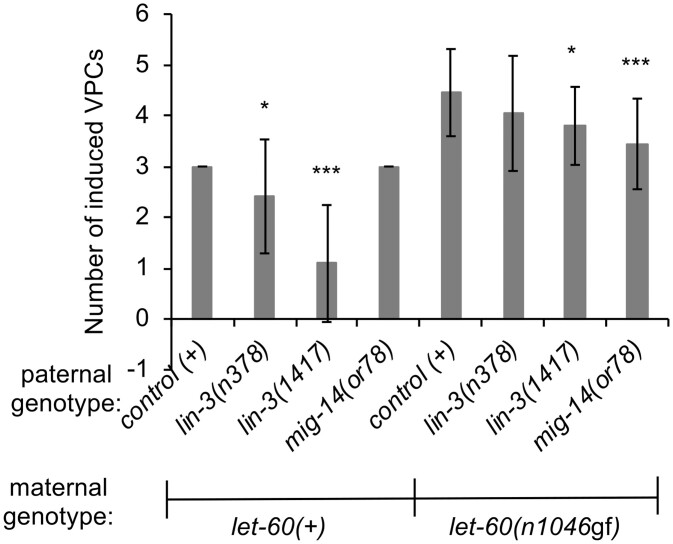
The nonautonomous modulators of *let-60(n1046*gf*)* act in a unique manner, distinct from known mesodermally derived signals that influence vulval development. Chimeric animals generated as in Fig. 3. VPC induction data for L4 chimeric animals, as in Fig. 4. Chimeric animals bearing reduction-of-function alleles of *lin-3/EGF* in P1 and *let-60(+)* in AB exhibit reduced production of vulval cell types. In animals with *let-60(n1046*gf*)* in AB, disruption of either *lin-3* or *mig-14* moderately reduces the number of induced VPCs. However, neither genotype exhibits an extensive reversion to wild type as observed with *hpo-18* or *szy-5* (Fig. 4). Error bars correspond to standard deviation. Asterisks indicate statistically different from control (*t*-test, *P < 0.05; ***P < 0.001). Full data and sample numbers provided in Supplementary tables.

Vulval development in *C. elegans* relies on signals external to the VPCs, including LIN-3/EGF from the AC (Hill and Sternberg 1992) and Wnt ligands (EGL-20, MOM-2, LIN-44, CWN-1, CWN-2), which are derived from multiple sources, including mesodermal cells (Harterink *et al.* 2011; Vargas-Velazquez *et al.* 2019). To ask whether knockdown of *hpo-18* or *szy-5* might modulate the phenotype in *let-60(n1046*gf*)* animals by altering the activity of any of these ligands, we evaluated chimeric animals with paternally contributed mutant alleles of *lin-3/egf* or *mig-14/wls*, which disrupts secretion of all Wnt ligands (Bänziger *et al.* 2006). In a *let-60(+)* background, mutant *lin-3* in the P1 lineage reduces vulval induction [Fig. 7; also observed in Artiles *et al.* (2019)]. When AB-derived cells are genotype *let-60(n1046gf)*, none of the *lin-3/egf* or *mig-14/wls* genotypes revert to wild type, as is observed for *hpo-18* or *szy-5* (Figs. 3 and 7). Thus, while this result does not rule out the possibility that loss of *hpo-18* or *szy-5* influences production or processing of LIN-3/EGF and Wnt ligands, it must alter additional processes or pathways beyond EGF or Wnt to produce the observed effect.

## Discussion

In this study we identified 24 *C. elegans* genes that promote the Muv phenotype elicited by hyperactive Ras (*let-60(n1046gf)*). The identified genes encode histones, ribosomes and mitochondrial ATP synthases, proteins involved in essential cell processes. While RNAi-mediated knockdown of the identified genes revert the *let-60(n1046*gf*)* phenotype to wild type, their knockdown has minimal effect in a *let-60(+)* genetic background, indicating that the effect is limited to the genetically perturbed mutant Ras condition and the genes do not generally impact normal development or function. In addition, these mesodermal gene functions are distinct from 2 mesodermal signaling classes of ligands (EGF and Wnt) known to influence VPC development, uncovering novel mechanisms in *C. elegans* that mediate intercellular communication between mesodermal cells and epithelial cells with an oncogenic form of Ras.

Several modifiers that block or revert the phenotype of *let-60(n1046*gf*)* in *C. elegans* have been identified previously, and such screens identified important components of the EGF-Ras-MAPK signaling pathway (Wu and Han 1994; Kornfeld *et al.* 1995; Singh and Han 1995; Sundaram and Han 1995). An important distinction between this and previous studies is that the RNAi knockdown (and the genetic disruption in chimeric animals) described here is limited to specific mesodermal-derived cells in the animals. This feature allows the screen to focus on genes that function outside of (rather than within) the VPCs and to identify essential genes like *hpo-18* and *szy-5* that, when subject to knockdown in the entire animal, would otherwise cause animals to die prior to vulval development. These genes are also distinct in that they may act less as pathway-specific modulators, and more in a gene (or allele) specific manner, since they influence the activity of activated alleles of *let-60/Ras*, but not the more upstream *let-23/EGFR*. Such allele-specific effects are similarly observed in cancer, where different oncogenic variants exhibit sensitivity to disruption of distinct inputs or effectors. For example, human tumors bearing KRasG13D are sensitive to EGFR inhibitors, whereas KRasG12D or G12V are not (De Roock *et al.* 2010; Rabara *et al.* 2019; Zafra *et al.* 2020; McFall and Stites 2021).

*szy-5* encodes a zinc finger protein, with 2 canonical triple-C2H2 domains (Wolfe *et al.* 2000; Iuchi 2001), 1 located near the amino terminus, and 1 near the carboxyl terminus. Although many canonical triple-C2H2 zinc finger proteins associate with specific DNA sequences to influence transcription (Emerson and Thomas 2009), these domains also mediate binding to RNA and interactions with other proteins (Hall 2005; Brayer and Segal 2008), so how SZY-5 impacts cell communication is as yet unclear. A temperature-sensitive, recessive mutant allele of the gene was initially identified as a Suppressor of *zyg-1*, a gene that encodes a kinase necessary for centrosome duplication (Kemp *et al.* 2007). In a *zyg-1(+)* background and at the restrictive temperature, these *szy-5* mutants also exhibit complex defects in microtubule arrangement, cytokinesis, chromosome segregation, and localization of the centrosome to the nuclear envelope (Kemp *et al.* 2007), suggestive of a role for SZY-5 in coordinating nuclear organization or functions. The identification here of several histone genes in similarly modulating phenotype in *let-60(n1046*gf*)* animals is consistent with a role for SZY-5 in impacting chromatin. There is extensive redundancy and sequence similarity among histone genes (Sural *et al.* 2020), which could underlie why RNAi knockdown (but not specific *his-71* mutations) is able to modulate the phenotype of *let-60(n1046*gf*)* animals.

This work also identifies a role for mitochondrial biology and ATP synthesis in 1 cell to impact the phenotype of another cell. Three genes encoding proteins of mitochondrial ATP complex V, the ATP synthase, were identified in the RNAi screen, *hpo-18*, *atp-5*, and *atp-4*. The cell nonautonomous functions of one of these genes, *hpo-18* (the ATP synthase F1ε gene), were genetically validated in chimeric animals using a mutant *hpo-18* allele. Other genes identified in the screen may also impact mitochondrial function and ATP production. Previous work shows that mitochondria are enriched in the basal half of the AC, and localized production of ATP can facilitate basement membrane breakdown between the AC and the developing vulval cells (Kelley *et al.* 2019), suggesting that some genes identified in this screen may be targeting an AC-specific process, but one that is distinct from known roles of EGF signaling in the AC. Decreasing mitochondrial ATP synthesis can increase reactive oxygen species, which have been shown to reduce the activity of *let-60(n1046*gf*)* (Murphy 2009; Kramer-Drauberg *et al.* 2020). However, this mechanism is interpreted to act in a cell-autonomous manner, whereas knockdown of the candidates from our screen suppress the Muv phenotype when knocked down in mesodermal cells, but not VPCs. Another possibility is that *hpo-18* is influencing the production of extracellular ATP, which is converted to adenosine and can signal to promote tumor cell proliferation and tumor progression, as well as modulate the activity of several cell types in the tumor microenvironment (Vultaggio-Poma *et al.* 2020; Antonioli *et al.* 2021). Future work will be required to determine how this fundamental cellular metabolite modulates activated Ras activity in a specific and cell nonautonomous manner.

Two of the 7 genes tested exhibited the same phenotype in the genetic chimera experiment as in the RNAi experiment. Differences in the genetic manipulation and the genetic background between the 2 types of experiments (genetic chimera analysis *vs.* an RNAi approach) likely explain why not all 7 candidate genes tested by chimera analysis modulate the *let-60(n1046*gf*)* phenotype. First, there is an incomplete overlap between the mesodermal cells affected by RNAi and P1-derived cells that contribute to chimeric animals. For example, a small subset of *myo-3*-expressing cells (the promoter used to restore *rde-1(+)* to muscle) are derived from AB (Fig. 3; Sulston *et al.* 1983; Ardizzi and Epstein 1987). In addition, only AB descendent cells harbor the *let-60(n1046*gf*)* or *let-60(n1700*gf*)* allele in chimeric animals, whereas all cells have the mutant allele in the RNAi strain. The mesodermal-RNAi strain also includes mutations that impact endogenous RNAi processing (*rde-1* and *rrf-3*) plus an integrated transgene incorporating multiple genes and plasmid sequences (Liu *et al.* 2017), which may produce a synthetic phenotype in combination with targeted genes. Second, there is the potential for maternal rescue in the chimeric animals. Despite the fact that vulval development occurs postembryonically during the third larval stage (L3), some genes that impact vulval development do exhibit maternal rescue (Ferguson and Horvitz 1989), and maternally provided products or epigenetic states may perdure into larval stages. Finally, our experimental assumptions with the RNAi approach may have technical limitations, including potential leakiness of the RNAi in other cell types (resulting in possible knockdown within the VPCs or other cells) and off-target RNAi effects. Similarly, the chimeric approach has some formal technical limitations, such as possible genetic adaptation induced by certain mutant alleles (Serobyan *et al.* 2020). While the functions of the other 5 genes tested by chimera experiments remain to be fully evaluated, here we emphasize that 2 very different approaches show a nonautonomous function for *hpo-18* and *szy-5* in promoting the ectopic cell proliferation phenotype conferred by *let-60(n1046gf)/Ras.* This demonstrates a novel, genotype-restricted role for these genes in mediating communication between mesodermal and epithelial cells.

## Supplementary Material

jkac200_Supplemental_Tables

jkac200_Supplemental_Figure_Legends

jkac200_Figure_S1

jkac200_Figure_S2

jkac200_Figure_S3

## Data Availability

All data are incorporated into the article and its online supplementary material. All *C. elegans* strains are available from the Caenorhabditis Genetics Center, or by contacting the corresponding author. Supplemental material is available at *G3* online.

## References

[jkac200-B1] Alexander J , Cukierman E. Cancer associated fibroblast: mediators of tumorigenesis. Matrix Biol. 2020;91–92:19–34. 10.1016/j.matbio.2020.05.004 PMC7434664 32450219

[jkac200-B2] Antonioli L , Fornai M, Pellegrini C, D'Antongiovanni V, Turiello R, Morello S, Haskó G, Blandizzi C. Adenosine signaling in the tumor microenvironment. Adv Exp Med Biol. 2021;1270:145–167. 10.1007/978-3-030&ndash;47189-7_9 33123998

[jkac200-B3] Ardizzi JP , Epstein HF. Immunochemical localization of myosin heavy chain isoforms and paramyosin in developmentally and structurally diverse muscle cell types of the nematode *Caenorhabditis elegans*. J Cell Biol. 1987;105(6 Pt 1):2763–2770. 10.1083/jcb.105.6.2763 3320053 PMC2114684

[jkac200-B4] Artiles KL , Fire AZ, Frøkjær-Jensen C. Assessment and maintenance of unigametic germline inheritance for *C. elegans*. Dev Cell. 2019;48(6):827–839.e9. 10.1016/j.devcel.2019.01.020 30799227 PMC6435406

[jkac200-B5] Bänziger C , Soldini D, Schütt C, Zipperlen P, Hausmann G, Basler K. Wntless, a conserved membrane protein dedicated to the secretion of Wnt proteins from signaling cells. Cell. 2006;125(3):509–522. 10.1016/j.cell.2006.02.049 16678095

[jkac200-B6] Beitel GJ , Clark SG, Horvitz HR. *Caenorhabditis elegans* ras gene let-60 acts as a switch in the pathway of vulval induction. Nature. 1990;348(6301):503–509. 10.1038/348503a0 2123303

[jkac200-B7] Brayer KJ , Segal DJ. Keep your fingers off my DNA: protein-protein interactions mediated by C2H2 zinc finger domains. Cell Biochem Biophys. 2008;50(3):111–131. 10.1007/s12013-008&ndash;9008-5 18253864

[jkac200-B8] Brügger MD , Valenta T, Fazilaty H, Hausmann G, Basler K. Distinct populations of crypt-associated fibroblasts act as signaling hubs to control colon homeostasis. PLoS Biol. 2020;18(12):e3001032. 10.1371/journal.pbio.3001032 33306673 PMC7758045

[jkac200-B9] Chamberlin HM , Jain IM, Corchado-Sonera M, Kelley LH, Sharanya D, et al Evolution of transcriptional repressors impacts Caenorhabditis vulval development. Mol Biol Evol. 2020;37(5):1350–1361. 10.1093/molbev/msaa009 PMC7182219 31960924

[jkac200-B10] De Roock W , Jonker DJ, Di Nicolantonio F, Sartore-Bianchi A, Tu D, Siena S, Lamba S, Arena S, Frattini M, Piessevaux H, et al Association of KRAS p.G13D mutation with outcome in patients with chemotherapy-refractory metastatic colorectal cancer treated with cetuximab. JAMA. 2010;304(16):1812–1820. 10.1001/jama.2010.1535 20978259

[jkac200-B11] Dickinson DJ , Pani AM, Heppert JK, Higgins CD, Goldstein B. Streamlined genome engineering with a self-excising drug selection cassette. Genetics. 2015;200(4):1035–1049. 10.1534/genetics.115.178335 26044593 PMC4574250

[jkac200-B12] Emerson RO , Thomas JH. Adaptive evolution in zinc finger transcription factors. PLoS Genet. 2009;5(1):e1000325. 10.1371/journal.pgen.1000325 19119423 PMC2604467

[jkac200-B13] Farhood B , Najafi M, Mortezaee K. Cancer-associated fibroblasts: secretions, interactions, and therapy. J Cell Biochem. 2019;120(3):2791–2800. 10.1002/jcb.27703 30260049

[jkac200-B14] Ferguson EL , Horvitz HR. The multivulva phenotype of certain *Caenorhabditis elegans* mutants results from defects in two functionally redundant pathways. Genetics. 1989;123(1):109–121. 2806880 10.1093/genetics/123.1.109 PMC1203774

[jkac200-B15] Hall TMT. Multiple modes of RNA recognition by zinc finger proteins. Curr Opin Struct Biol. 2005;15(3):367–373. 10.1016/j.sbi.2005.04.004 15963892

[jkac200-B16] Harterink M , Kim DH, Middelkoop TC, Doan TD, van Oudenaarden A, Korswagen HC. Neuroblast migration along the anteroposterior axis of *C. elegans* is controlled by opposing gradients of Wnts and a secreted Frizzled-related protein. Development. 2011;138(14):2915–2924. 10.1242/dev.064733 21653614 PMC3119304

[jkac200-B17] Hill RJ , Sternberg PW. The gene lin-3 encodes an inductive signal for vulval development in *C. elegans*. Nature. 1992;358(6386):470–476. 10.1038/358470a0 1641037

[jkac200-B18] Iber D. The control of lung branching morphogenesis. Curr Top Dev Biol. 2021;143:205–237. 10.1016/bs.ctdb.2021.02.002 33820622

[jkac200-B19] Ihara S , Hagedorn EJ, Morrissey MA, Chi Q, Motegi F, Kramer JM, Sherwood DR. Basement membrane sliding and targeted adhesion remodels tissue boundaries during uterine-vulval attachment in *Caenorhabditis elegans*. Nat Cell Biol. 2011;13(6):641–651. 10.1038/ncb2233 21572423 PMC3107347

[jkac200-B20] Iuchi S. Three classes of C2H2 zinc finger proteins. Cell Mol Life Sci. 2001;58(4):625–635. 10.1007/PL00000885 11361095 PMC11146492

[jkac200-B21] John A , Rauzi M. A two-tier junctional mechanism drives simultaneous tissue folding and extension. Dev Cell. 2021;56(10):1469–1483.e5. 10.1016/j.devcel.2021.04.003 33891900

[jkac200-B22] Jose AM , Hunter CP. Transport of sequence-specific RNA interference information between cells. Annu Rev Genet. 2007;41:305–330. 10.1146/annurev.genet.41.110306.130216 17645412 PMC7377521

[jkac200-B23] Kamath RS , Fraser AG, Dong Y, Poulin G, Durbin R, Gotta M, Kanapin A, Le Bot N, Moreno S, Sohrmann M, et al Systematic functional analysis of the *Caenorhabditis elegans* genome using RNAi. Nature. 2003;421(6920):231–237. 10.1038/nature01278 12529635

[jkac200-B24] Katz WS , Lesa GM, Yannoukakos D, Clandinin TR, Schlessinger J, Sternberg PW. A point mutation in the extracellular domain activates LET-23, the *Caenorhabditis elegans* epidermal growth factor receptor homolog. Mol Cell Biol. 1996;16(2):529–537. 10.1128/MCB.16.2.529 8552080 PMC231031

[jkac200-B25] Kelley LC , Chi Q, Cáceres R, Hastie E, Schindler AJ, Jiang Y, Matus DQ, Plastino J, Sherwood DR. Adaptive F-actin polymerization and localized ATP production drive basement membrane invasion in the absence of MMPs. Dev Cell. 2019;48(3):313–328.e8. 10.1016/j.devcel.2018.12.018 30686527 PMC6372315

[jkac200-B26] Kemp CA , Song MH, Addepalli MK, Hunter G, O'Connell K. Suppressors of zyg-1 define regulators of centrosome duplication and nuclear association in *Caenorhabditis elegans*. Genetics. 2007;176(1):95–113. 10.1534/genetics.107.071803 17446307 PMC1893046

[jkac200-B27] Kornfeld K , Guan KL, Horvitz HR. The *Caenorhabditis elegans* gene mek-2 is required for vulval induction and encodes a protein similar to the protein kinase MEK. Genes Dev. 1995;9(6):756–768. 10.1101/gad.9.6.756 7729691

[jkac200-B28] Kramer-Drauberg M , Liu J-L, Desjardins D, Wang Y, Branicky R, Hekimi S. ROS regulation of RAS and vulva development in *Caenorhabditis elegans*. PLoS Genet. 2020;16(6):e1008838. 10.1371/journal.pgen.1008838 32544191 PMC7319342

[jkac200-B29] Liu H , Dowdle JA, Khurshid S, Sullivan NJ, Bertos N, Rambani K, Mair M, Daniel P, Wheeler E, Tang X, et al Discovery of stromal regulatory networks that suppress Ras-sensitized epithelial cell proliferation. Dev Cell. 2017;41(4):392–407.e6. 10.1016/j.devcel.2017.04.024 28535374 PMC5508591

[jkac200-B30] McFall T , Stites EC. Identification of RAS mutant biomarkers for EGFR inhibitor sensitivity using a systems biochemical approach. Cell Rep. 2021;37(11):110096. 10.1016/j.celrep.2021.110096 34910921 PMC8867612

[jkac200-B31] Murphy MP. How mitochondria produce reactive oxygen species. Biochem J. 2009;417(1):1–13. 10.1042/BJ20081386 19061483 PMC2605959

[jkac200-B32] Nelson CM , Bissell MJ. Of extracellular matrix, scaffolds, and signaling: tissue architecture regulates development, homeostasis, and cancer. Annu Rev Cell Dev Biol. 2006;22:287–309. 10.1146/annurev.cellbio.22.010305.104315 16824016 PMC2933192

[jkac200-B33] Paulsson J , Micke P. Prognostic relevance of cancer-associated fibroblasts in human cancer. Semin Cancer Biol. 2014;25:61–68. 10.1016/j.semcancer.2014.02.006 24560651

[jkac200-B34] Qadota H , Inoue M, Hikita T, Köppen M, Hardin JD, Amano M, Moerman DG, Kaibuchi K. Establishment of a tissue-specific RNAi system in *C. elegans*. Gene. 2007;400(1–2):166–173. 10.1016/j.gene.2007.06.020 17681718 PMC3086655

[jkac200-B35] Rabara D , Tran TH, Dharmaiah S, Stephens RM, McCormick F, Simanshu DK, Holderfield M. KRAS G13D sensitivity to neurofibromin-mediated GTP hydrolysis. Proc Natl Acad Sci U S A. 2019;116(44):22122–22131. 10.1073/pnas.1908353116 31611389 PMC6825300

[jkac200-B36] Rasband WS. ImageJ. Bethesda (MD, USA): National Institutes of Health; 1997.

[jkac200-B37] Reiner DJ , González-Pérez V, Der CJ, Cox AD. Use of *Caenorhabditis elegans* to evaluate inhibitors of Ras function in vivo. Methods Enzymol. 2008;439:425–449. 10.1016/S0076-6879(07)00430-2 18374181

[jkac200-B38] Sawin ER , Ranganathan R, Horvitz HR. *C. elegans* locomotory rate is modulated by the environment through a dopaminergic pathway and by experience through a serotonergic pathway. Neuron. 2000;26(3):619–631. 10.1016/s0896-6273(00)81199-x 10896158

[jkac200-B39] Serobyan V , Kontarakis Z, El-Brolosy MA, Welker JM, Tolstenkov O, Saadeldein AM, Retzer N, Gottschalk A, Wehman AM, Stainier DY, et al Transcriptional adaptation in *Caenorhabditis elegans*. eLife. 2020;9:e50014. 10.7554/eLife.50014 31951195 PMC6968918

[jkac200-B40] Simmer F , Tijsterman M, Parrish S, Koushika SP, Nonet ML, Fire A, Ahringer J, Plasterk RHA. Loss of the putative RNA-directed RNA polymerase RRF-3 makes *C. elegans* hypersensitive to RNAi. Curr Biol. 2002;12(15):1317–1319. 10.1016/s0960-9822(02)01041-2 12176360

[jkac200-B41] Singh N , Han M. sur-2, a novel gene, functions late in the let-60 ras-mediated signaling pathway during *Caenorhabditis elegans* vulval induction. Genes Dev. 1995;9(18):2251–2265. 10.1101/gad.9.18.2251 7557379

[jkac200-B42] Sternberg PW , Han M. Genetics of RAS signaling in *C. elegans*. Trends Genet. 1998;14(11):466–472. 10.1016/s0168-9525(98)01592-3 9825675

[jkac200-B43] Stiernagle T. Maintenance of *C. elegans*. WormBook. 2006;1–11. 10.1895/wormbook.1.101.1 PMC4781397 18050451

[jkac200-B44] Sulston JE , Schierenberg E, White JG, Thomson JN. The embryonic cell lineage of *the nematode Caenorhabditis elegans*. Dev. Biol. 1983;100(1):64–119. 10.1016/0012&ndash;1606(83)90201-4 6684600

[jkac200-B45] Sundaram M , Han M. The *C. elegans* ksr-1 gene encodes a novel Raf-related kinase involved in Ras-mediated signal transduction. Cell. 1995;83(6):889–901. 10.1016/0092&ndash;8674(95)90205-8 8521513

[jkac200-B46] Sural S , Liang C-Y, Wang F-Y, Ching T-T, Hsu A-L. HSB-1/HSF-1 pathway modulates histone H4 in mitochondria to control mtDNA transcription and longevity. Sci Adv. 2020;6:eaaz4452. 10.1126/sciadv.aaz4452 33087356 PMC7577724

[jkac200-B47] Tabara H , Sarkissian M, Kelly WG, Fleenor J, Grishok A, Timmons L, Fire A, Mello CC. The rde-1 gene, RNA interference, and transposon silencing in *C. elegans*. Cell. 1999;99(2):123–132. 10.1016/s0092-8674(00)81644-x 10535731

[jkac200-B48] Vargas-Velazquez AM , Besnard F, Félix M-A. Necessity and contingency in developmental genetic screens: EGF, Wnt, and Semaphorin pathways in vulval induction of the nematode Oscheius tipulae. Genetics. 2019;211(4):1315–1330. 10.1534/genetics.119.301970 30700527 PMC6456316

[jkac200-B49] Vultaggio-Poma V , Sarti AC, Virgilio FD. Extracellular ATP: A Feasible Target for Cancer Therapy. Cells. 2020;9(11):2496. 10.3390/cells9112496 PMC7698494 33212982

[jkac200-B50] Wolfe SA , Nekludova L, Pabo CO. DNA recognition by Cys2His2 zinc finger proteins. Annu Rev Biophys Biomol Struct. 2000;29:183–212. 10.1146/annurev.biophys.29.1.183 10940247

[jkac200-B51] Wu Y , Han M. Suppression of activated Let-60 ras protein defines a role of *Caenorhabditis elegans* Sur-1 MAP kinase in vulval differentiation. Genes Dev. 1994;8(2):147–159. 10.1101/gad.8.2.147 8299935

[jkac200-B52] Zafra MP , Parsons MJ, Kim J, Alonso-Curbelo D, Goswami S, Schatoff EM, Han T, Katti A, Fernandez MTC, Wilkinson JE, et al An in vivo KRAS allelic series reveals distinct phenotypes of common oncogenic variants. Cancer Discov. 2020;10(11):1654–1671. 10.1158/2159&ndash;8290.CD-20&ndash;0442 32792368 PMC7642097

