# Supplementary material for: Discovery of nonautonomous modulators of activated Ras
Source: G3 (Bethesda). 2022 Aug 5;12(10):jkac200. doi: 10.1093/g3journal/jkac200 (PMC9526067; doi:10.1093/g3journal/jkac200)
Supplement: jkac200_Supplemental_Figure_Legends [file jkac200_supplemental_figure_legends.docx]

**Supplemental Figure Legends.**

**Supplemental Figure 1. Extended images for Figure 2.** (A-F) Uncropped DIC images of the ventral epidermis in L4 stage, as represented in Figure 2 (A-F). A.  In wild-type animals, the offspring of three ventral epithelial cells (vulval precursor cells, VPCs) organize to form a single vulval opening, identified with a white arrow, as in Figure 1. B. *let-60(n1046*gf*)* mutants bearing the mesodermal-RNAi system, treated with control RNAi, produce a Multivulva (Muv) phenotype with a primary vulval opening (white arrow) as well as ectopic vulval tissue (black arrow) resulting from the division of additional VPCs. (C-F) Images of mesodermal-RNAi knockdown of representative genes, illustrating the reversion of *let-60(n1046*gf*)* to a wild-type phenotype.

**Supplemental Figure 2. RNAi knockdown of candidate genes within the VPCs does not revert the Muv phenotype of let-60(*n1046*gf).** Most non-wild-type animals are Muv. Data presented as percent wild type for best comparison to data in Figure 2. n ≥ 40 for each condition. Error bars correspond to standard error. Full data and sample numbers provided in supplemental data tables.

**Supplemental Figure 3. Mitochondria localization in the anchor cell is maintained upon mesodermal-RNAi knockdown of genes.** Hermaphrodite animals from CM2453 (mesodermal-RNAi strain) were treated with RNAi under conditions that produce a phenotype and selected as L3s and stained with MitoTracker Red CMXRos to visualize mitochondria. (A-F) Under wild type conditions, mitochondria are enriched near the basolateral surface of the anchor cell (white arrows indicate anchor cell) to supply ATP to newly-formed invadopodia extending towards the underlying VPCs (black and white brackets). Staining appears unchanged in animals treated with RNAi targeting *hpo-18* or *szy-5* (G) Quantification of the effect. We observed no difference in mitochondria enrichment in control compared to experimental conditions. Polarity represents the ratio of basal to apical fluorescence (see Materials and Methods). n = 10 for each RNAi knockdown. Horizontal lines indicate average values.
