# Supplementary figures and images for: Discovery of nonautonomous modulators of activated Ras
Source: G3 (Bethesda). 2022 Aug 5;12(10):jkac200. doi: 10.1093/g3journal/jkac200 (PMC9526067; doi:10.1093/g3journal/jkac200)

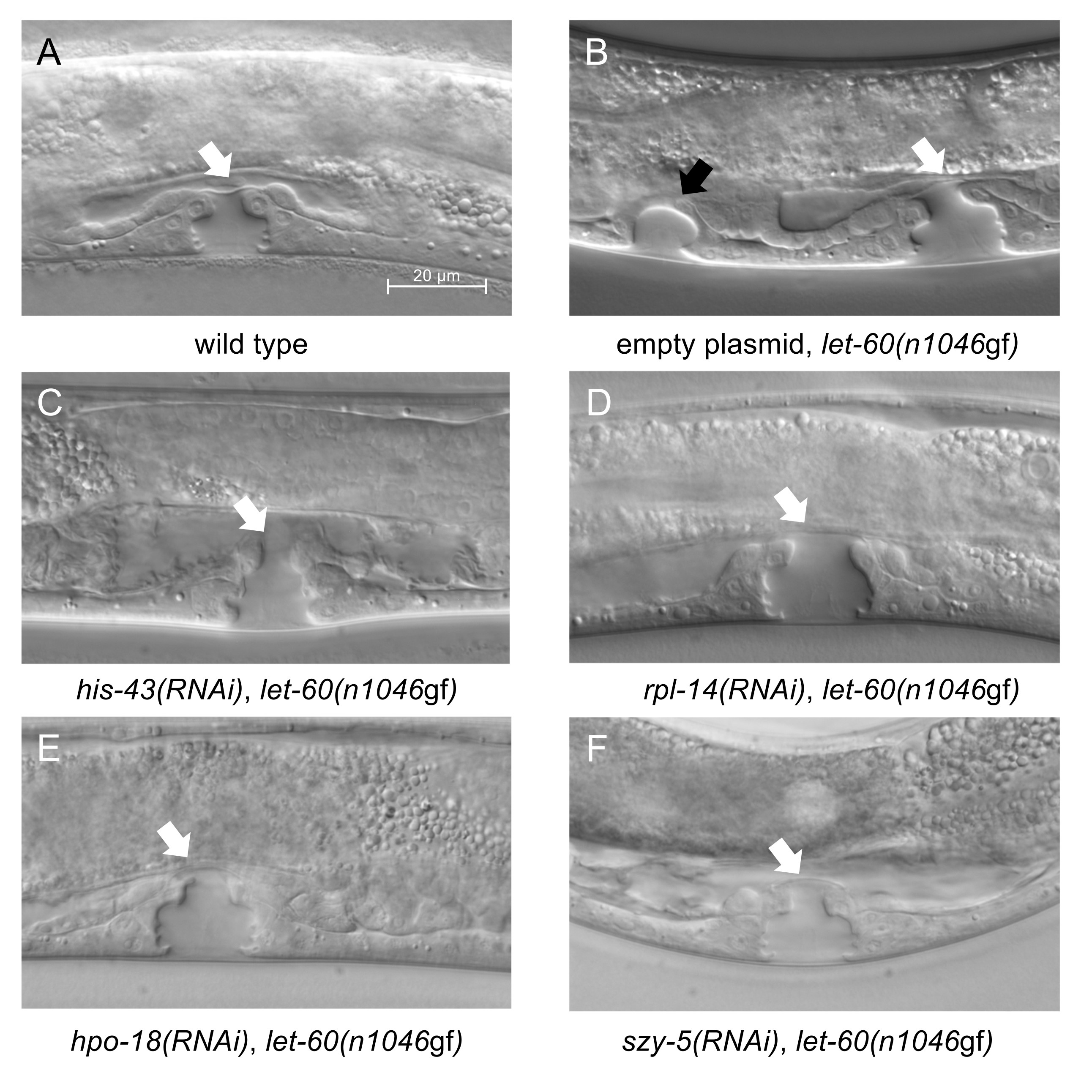

Supplement: jkac200_Figure_S1 [file jkac200_figure_s1.jpeg]

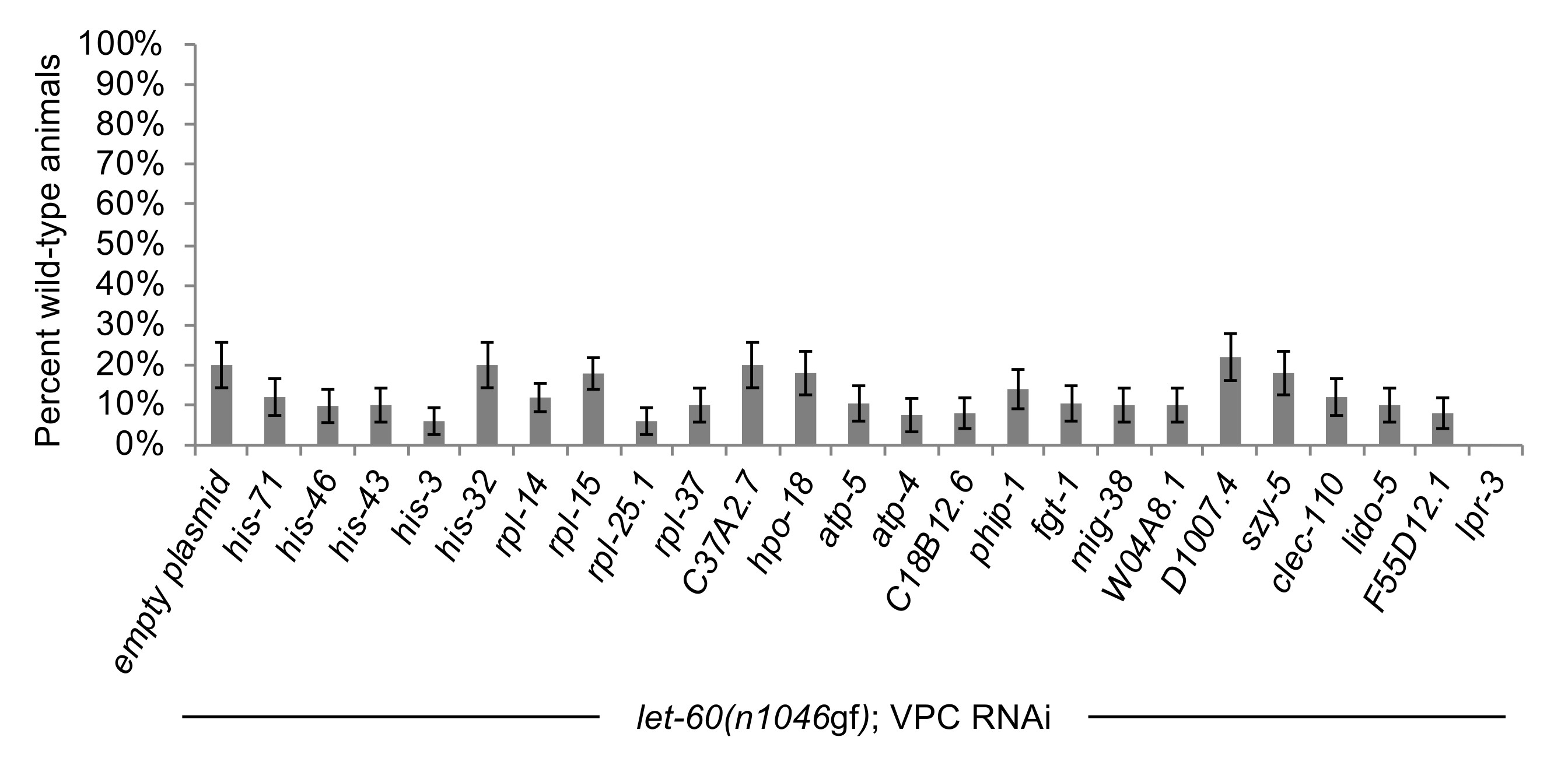

Supplement: jkac200_Figure_S2 [file jkac200_figure_s2.jpeg]

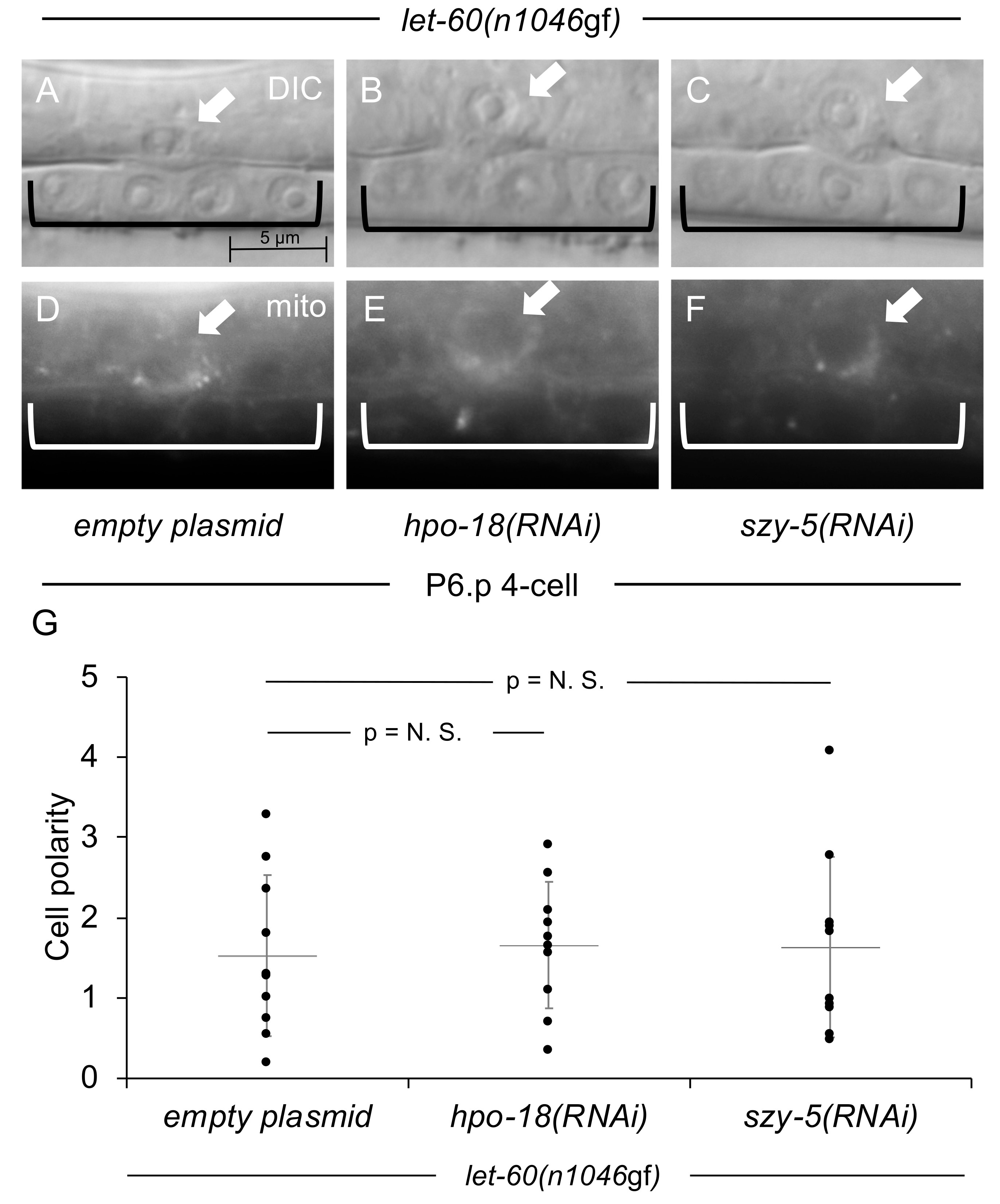

Supplement: jkac200_Figure_S3 [file jkac200_figure_s3.jpeg]
